# Spring-Block Model Reveals Region-Like Structures

**DOI:** 10.1371/journal.pone.0016518

**Published:** 2011-02-08

**Authors:** Gabriell Máté, Zoltán Néda, József Benedek

**Affiliations:** 1 Department of Theoretical and Computational Physics, Babeş-Bolyai University, Cluj-Napoca, Romania; 2 Institute for Theoretical Physics, Heidelberg University, Heidelberg, Germany; 3 Department of Human Geography, Babeş-Bolyai University, Cluj-Napoca, Romania; University of Maribor, Slovenia

## Abstract

A mechanical spring-block model is used for realizing an objective space partition of settlements from a geographic territory in region-like structures. The method is based on the relaxation-dynamics of the spring-block system and reveals in a hierarchical manner region-like entities at different spatial scales. It takes into account in an elegant manner both the spatiality of the elements and the connectivity relations among them. Spatiality is taken into account by using the geographic coordinates of the settlements, and by detecting the neighbors with the help of a Delaunay triangulation. Connectivity between neighboring settlements are quantified using a Pearson-like correlation for the relative variation of a relevant socio-economic parameter (population size, GDP, tax payed per inhabitant, etc.). The method is implemented in an interactive JAVA application and it is applied with success for an artificially generated society and for the case of USA, Hungary and Transylvania.

## Introduction

The classical methods and models of exact sciences proved to be many times useful and inspiring in tackling social science problems. For a long time social scientist were reticent and had an incredulous attitude towards the applicability of such methods and models, considering them in the best case as simple metaphors [1]. Probably the first one who have taken seriously the possibility of applying the methods of physics in describing social phenomena is the Belgian statistician Adolphe Quetelet [2], who stated that as the number of individuals or entities in the system grows, the individualities becomes less important and one can approach the system with models borrowed from physics. He named this approach “social physics”. These type of approaches has been long debated, there are many arguments pro and contra, and nowadays still many social scientist cannot take seriously physical models for describing social phenomena [3], [4]. Beside all critics from social sciences, a considerable part of the statistical physics society persists in considering its models for describing social phenomena. Stories of success are the nowadays acclaimed econophysics [5] and network science [6], [7]. The work presented here is a social physics attempt for a major and old problem in the field of social sciences, namely the objective partition of a geographical territory in regions.

Partitioning settlements of a geographic territory in smaller parts is a problem of economic, political and social relevance. Regions are such structures and are regarded by a large number of authors [8]–[10] as basic units of the social and economic life. Regions do not have a rigorous definition and there are still two basic and much debated questions that should be clarified: how do they emerge and how may be defined and delimited in space? Regions can be defined also at different spatial scales which also adds an extra complexity to these basic questions. Irrespective of whether the region is regarded as a social construct or as an entity with material existence, the question of the border emerges, that is: how is a region delimited? The situation is easier if the borders are present normatively: political borders (states) and administrative borders (sub national level). Homogeneous regions are easy to define by selecting of one or more statistic variables, than by grouping their values into classes, and finally by mapping the borders of the classes. Substantial difficulties emerge however at the delimitation of regions that are regarded as social constructs, the central category of which is the identity. Much focus is on the delimitation of the functional regions, defined on the base of the frequency, intensity and spatial orientation of the intra-regional interactions [11], [12]. Regardless of the method applied, regions are to a certain extent methodological constructs. This means that their precise borderline is determined by the chosen delimitation method.

We see thus that regions are complex structures, and in order to detect them complex methods are needed. Both the spatiality and the connectivity of the involved settlements has to be taken into account when one wants to draw region-borders. Spatiality appears in the problem as neighboring relations among the settlements and as spatial scales, while the concept of connectivity is a complex notion which can be quantifiable or non-quantifiable. There are many measures or attributes to characterize connectivity of the settlements, reflecting also the similarity of the population in these settlements. These measures can have also different importance or weights in the global evaluation of a unique concept which determines the connection strength among them.

In human-geography there are many protocols for defining regions, most of them being rather subjective [12]–[15]. The need for an objective partition of a geographical territory stands for a long time and the social physics approach has already a long history in this field.

The most straightforward approach is based on a geometrical construction, namely the classical space-tessellation (Voronoi cells), constructed around the a priori detected centers [16], [17]. This type of approach overestimates the role of spatiality and neglects totally the similarity and the socio-economic connections among the settlements. Also, the centers are usually rather arbitrarily defined. In the same lines, but a more physical approach is the one based on the “gravitational model” [18]–[20]. This model exploits an analogy with Newtonian gravity for defining attraction basins. Settlements are considered as point-like masses, their sizes are interpreted as a mass, and their spatial distances are the distances in the Newtonian gravitation law. Within this approach, besides the spatial distribution, the size-distribution of the settlements is also taken into account for defining the relevant space partitioning. The “potential model” [19]–[22], generalizes further the analogy with an interacting particle system. It defines a kind of generalized potential composed of several terms in each point of the considered space. From this potential field in analogy with classical fields in physics a vector force-field is derived, which governs the flow of particles in the space. Through this force-field the potential model is useful for detecting attraction basins and consequently to delimit regions. Recently the networks view gained also importance in modeling and approaching complex systems. Revealing and analyzing hierarchical settlements, money-flow, telecommunication or transportation networks [15], [23]–[25] is also a viable approach to construct regions at different scales. Recent studies offered great perspectives in such sense [23], [24].

In the present work we intend to present another simple and objective space-partition method, based on a classical model of physics: namely the spring-block system. The model takes into account both the spatiality and the connectivity strength of settlements in order to detect regions. It offers a simple and visual way to incorporate these concepts in a classical physics model. The idea of using spring-block type models comes from the fact that such models were used with success to describe fragmentation at different spatial scales [26]. The model uses sliding blocks interconnected by springs as main elements. The blocks will model the settlements, interconnected with their neighbors through abstracts springs. The mass of the blocks are naturally the sizes of the settlements, while the interaction strength in the springs is defined through the connectivity strength of the neighboring elements. This measure will be determined from Pearson-type correlations of a relevant long-time settlement-level data (tax, income, population size, etc.). The clusters which result from the relaxation of the tension-field in the spring-block system are accounted as regions of the corresponding geographical system.

## Methods

### The spring-block system

**Figure 1:**
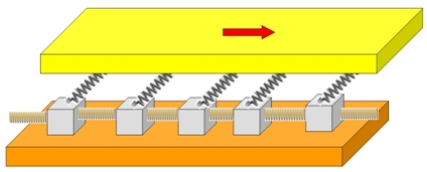
Main elements of the one-dimensional Burridge-Knopoff model. The blocks are connected to each other and to the upper plane by springs. The upper plane is dragged with a constant velocity. Since the blocks are allowed to slide, they will slide in avalanches following the motion of the upper surface.

Spring-block type models were first introduced by R. Burridge and L. Knopoff in 1967 [27] for explaining the empirical law of Gutenberg and Richter on the size distribution of the earthquakes. The model consists of simple units: blocks interconnected by springs which are allowed to slide with friction on a plane. The involved tectonic plates were modeled by two surfaces interconnected through a chain of sliding blocks (Figure 1). The upper plane (to which the blocks are connected by springs) is dragged with a constant velocity. As a result of this the blocks will slide in avalanches following the motion of the upper surface. The breakthrough of the model was that the energy dissipation in the avalanches exhibited a power-law distribution in agreement with the empirical law of Gutenberg and Richter. The model was generalized in two dimensions by Olami, Feder and Christensen [28]. Due to the exponential boom in computational power and advances in computer simulation techniques, the model gained popularity and it was used for modeling a great variety of physical phenomena. Known examples in this respect are the PLC (Portevin-Le Chatelier) phenomena [29], structures formed by the capillary self-organization of nanoparticle systems [30] magnetization phenomena and Barkhausen noise [31] or even highway traffic [32].

**Figure 2:**
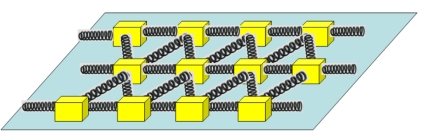
The two-dimensional spring-block system used for modeling quasi-static fragmentation phenomena. The blocks are interconnected by springs and they can slide on a two-dimensional substrate.

The present work is motivated by the use of the spring-block system in modeling the quasi-static fragmentation which occurs in drying granular materials in contact with a frictional substrate [26], [33]. Examples from everyday life for such phenomenon are the drying of mud, clay or dye, which leads to the well-known and characteristic polygonal fragment structures. The spring-block approach for such a phenomenon models the grains of the material by blocks which can slide on a two-dimensional substrate. The capillarity effect of the drying fluid is modeled by springs that connect the blocks [26]. Initially the blocks are displaced on a triangular lattice on a 2D substrate and neighboring blocks are connected by springs that are randomly stressed (Figure 2). Drying is modeled as a relaxation process. During each relaxation step we allow the blocks to slide to a new equilibrium position and each spring has the possibility to break. Block sliding will occur when the total force acting on it is greater than the friction force and a spring will break if the tension in it exceeds a breaking threshold. In each simulation step the system is relaxed. This means that all blocks will be moved until the resultant spring forces acting on them are smaller than the friction force and all spring are broken until the tensions in the remained ones are smaller than the breaking threshold. After relaxation the spring-tensions are consecutively increased (modeling the build up of stresses due to evaporation) and relaxation dynamics is imposed again. Due to the competing effects of the spring tensions and frictional forces, blocks will slide in avalanches leading finally to the breakage of the springs and thus to fragmentation of the system. The above presented spring-block model yields fragmentation patterns in good agreement with the experimentally observed ones.

### Spring-block system applied for space-partition

We apply now the spring-block system and a relaxation type dynamics for detecting regions on different length-scales. The model used for quasi-static fragmentation can be implemented with some modifications.

#### Blocks

Settlements in a given geographical territory will be regarded as point-like blocks of different sizes. The size of the settlement (population) will determine the mass of the corresponding block. The blocks are displaced on a plane (surface), respecting the relative geographical position of the settlements they represent. A 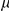 friction coefficient is imposed for characterizing the friction between the blocks and the surface. This means that the static friction force acting on each block 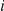 is
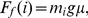
(1)where for the sake of simplicity we define the gravitational constant as 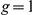, giving the units for the forces.

The blocks are connected with their neighbors by abstract springs.

#### Neighbors

**Figure 3:**
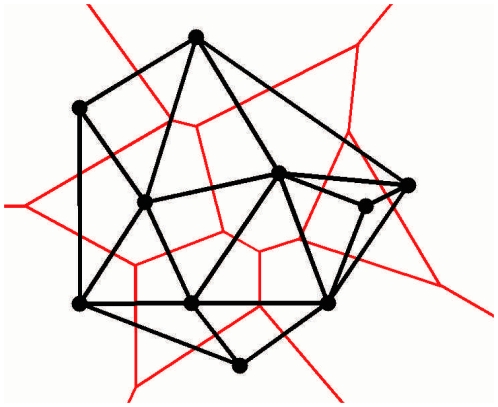
Voronoi tessellation for determining the neighboring relation of the blocks. The red lines are the segments of the Voronoi tessellation, the black ones are the edges of the Delaunay graph (triangulation).

Before discussing the spring forces, let us first clarify what we mean by and how we detect neighbors. Since the blocks are not following any regular lattice structure, the most straightforward way to detect neighbors are by considering a Voronoi tessellation [34] and detecting neighboring cells. By definition, the Voronoi tessellation of the set of points 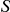 is a tessellation in which each Voronoi cell 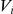 corresponding to the site 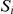 consists of all the points of the space closer to 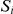 than any other site. The segments of the Voronoi diagram are all the points in the plane that are equidistant to sites corresponding to neighboring Voronoi cells. The Voronoi tessellation can be transformed in the Delaunay triangulation by connecting the sites of the neighboring Voronoi cells. Two settlements (blocks) will be considered neighbors if their corresponding Voronoi cells are neighbors (or if they are directly connected by the Delaunay triangulation). In Figure 3 we illustrate the Voronoi tessellation method for detecting the neighbors.

#### Springs

Once the neighboring relations are defined the next step is to connect neighbors by abstract springs. These springs will attract settlements that are connected in socio-economic sense. In order to have the tensions in the springs some sort of connectivity measure has to be defined, and after that we have to give the explicit distance dependence of this interaction force. Naturally, settlements that are more associated has to attract each-other more strongly. In the same line of thinking, settlements that are closer are more likely to be in the same regions, so the force in the springs has to be inversely proportional with the distance between the connected neighbors. Consequently the force acting on element 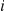 connected by element 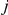 writes as:
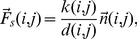
(2)The term 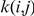 stands for the connectivity strength between neighboring settlements 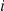 and 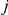, 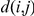 is the distance between settlements 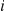 and 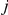, and 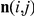 is the unit vector oriented the same as the line which connects block 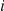 and 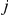 and directed from element 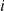 to 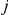. This means that the force in the abstract springs are not harmonic, and the analogy with classical springs has to be used with care! The notion of springs is however still justified, since this interaction connects only two specified particles, like springs do.

#### The Connectivity Measure

In order to have the explicit values of the spring forces, we still need to define a measure of connectivity, which is the driving force in the course-graining mechanism. Let us consider that there is settlement-level data for a relevant socio-economic quantity on a relatively long time-period. This could be for instance the total population, GDP, tax per inhabitant, etc… and denote this quantity for settlement 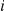 at time instance 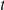 as 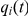. Let us assume that the time-sampling is uniform an the time-steps for the sampling are considered as unit times. It is possible to define than the relative change of this quantity for the consecutive time-steps as:
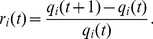
(3)


Our main hypothesis for defining the connectivity measure is that for settlements that are socio-economically connected the time-like variation of the 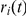 values has to be correlated. In a mathematical notation, our hypothesis writes
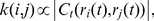
(4)where 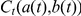 denotes a time-like correlation measure between the time-dependent quantities 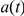 and 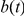. The simplest way to obtain this correlation measure is by using the Pearson correlation
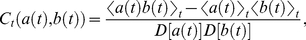
(5)where we denoted by 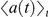 the time average of quantity 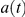 and by 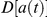 it's standard deviation.For simplicity we take the proportionality constant in the relation between 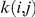 and 
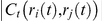
 as unity, thus equation (4) will be written as equality. Please also note, that a strong negative correlation (an anti-correlation) is also a sign of a strong connectivity, this is the reason why in eq. (4) we use the modulus.

Once the elements of the spring-block model are clarified, we define the dynamics of the model and describe how the dynamics will partition the settlements in groups of various sizes.

#### The Dynamics

Initially the blocks representing the settlements are displayed in the simulation plane, respecting the GPS coordinates of the settlements. Neighbors are than identified by a Voronoi construction and are connected through abstract springs. The tensions in the springs are calculated using equation (2) and (4). The system is thus pre-tensioned and the dynamics of the system is a classical relaxation dynamics. In contrast with the models used for fragmentation, here the springs do not have to be broken in order to obtain a course-graining because the 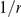 nature of the tension forces will group the elements anyhow.

**Figure 4:**
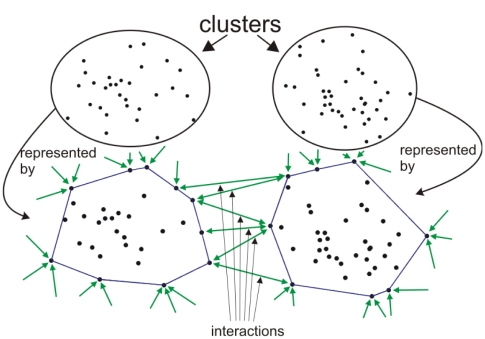
Coarse-graining in the spring-block approach. The figure illustrates how the links are inherited when blocks juncture.

The dynamics of the system is driven by the 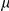 friction coefficient. At each simulation time-step we calculate for each element 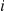 the resultant force 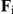 acting from the attached springs:
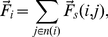
(6)

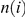
 denoting the set of neighbors of element 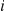. If the magnitude of this force is bigger than the friction force, block 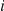 is allowed to move in the direction of 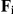. The displacement in each simulation step is taken as a 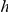 constant, independently of the magnitude of the resultant force. We considered thus an over-damped relaxation. If block 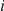 and 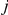 comes closer than a 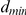 value they will be glued together and therefore will be represented by a new block with mass 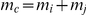. This new block will inherit all the links of blocks 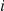 and 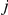 (see Figure 4). We define now settlement clusters as groups that are represented by the same block.

Initially 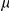 is set to a large value so that no block can move. We then decrease 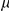 until the first block is allowed to slide. The sliding of the block will reorder the system and can induce further moves or juncture. We iteratively relax the system handle collisions and slides until we have fulfilled for each block 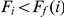. The value of the 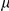 friction coefficient is recursively lowered until the desired partition (number of clusters) is reached.

The above algorithm will group the elements respecting their connection strength with neighbors and the initial spatiality. Smaller blocks will slide towards large ones, since the friction forces acting on the latter ones are larger. These large settlements will than naturally become the centers of the clusters. It is then reasonable to assume that the spring-block relaxation dynamics will reveal regions at different scales, as the system hierarchically form larger and larger clusters.

The model as it is defined here has only two adjustable parameters: the value of the sliding step 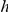 and minimal allowed distance 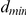. Assuming the value of the sliding step small enough 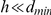, it's chosen value will not influence the obtained cluster structure. In the same manner, taking the value of 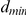 smaller than the smallest distance in the original configuration is enough to ensure that the final cluster structure is stable, and is not influenced by the choice of it.

The method and model described above was implemented in an interactive JAVA application [35], which allows the user to follow visually the whole clustering process up to the end when all blocks concur in one. The program memorizes all the intermediary situations and can interactively visualize the settlement partitions corresponding to them.

## Results

### Testing the method on a model system

**Figure 5:**
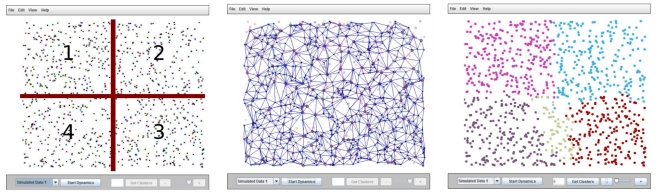
Testing the spring-block system on a model situation. As shown in the figure on the left four domains are defined so that connectivity inside the domains are stronger than connectivity between the domains. The picture in the middle shows an intermediate simulation step, with the existing springs and blocks. The figure on the right shows the detected partition.

In order to prove that the method works, we first consider a model system with settlements that have randomly generated initial asset (denoted by 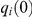), mass (size/population) and position inside a square-like domain. The assets and masses are generated according to a normal distribution (positive part of it) and they are proportional to each other. Our hypothesis here is that in a real world case the initial asset of a settlement should be proportional to the size of the settlement, meaning that everybody contributes to the total asset with the same amount. The positions of the settlements are randomly distributed using a uniform distribution. Four clusters are defined by dividing the domain in four equal parts as shown in Figure 5. An asset-exchange model [36] is then imposed on this system with the constraint that transactions between settlements are more likely inside the pre-defined clusters and the likelihood for having a transaction decreases as the distance between the settlements increases. If 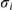 denotes the cluster in which settlement 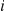 is (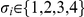), 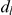 is the diagonal length of the square-like area, 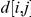 is the distance between settlements 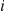 and 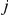 our asset-exchange dynamics has the following steps:

1. pick randomly two settlements, let these two settlements be denoted by 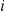 and 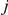

2. accept the choice with probability
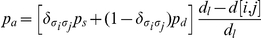
with the condition that 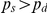

3. if the pair 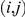 is accepted we transact a mart between them:

- a fair one with 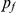 probability:
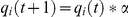


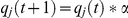


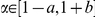
 uniformly distributed random number, where the parameters 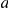 and 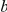 satisfy: 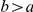, 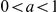, that is, the probability of gain is larger then the probability of loss
- an unfair one with 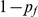 probability:
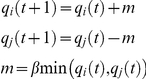
with 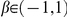 uniformly distributed random number

The parameters were selected as: 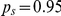, 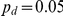, 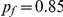, 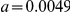, 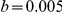. Note that with the given values 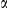 is always close to 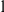. This should be the case because otherwise the gains or losses in fair transactions would be unreally large and the changes in the assets would be much too random killing all correlations.

First we make 1000 asset-exchange steps for thermalizing the system, then we start to record the assets after each asset-exchange step. The consecutively recorded assets will be considered as the relevant 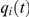 data needed in our method. We apply now our method using this data and the randomly generated coordinates.

In Figure 5 we illustrate the starting configuration of the spring-block system, an intermediate one with the connecting springs, and the partition that reveals the imposed connectivity. It can be observed that the program detects the imposed structure if we look for a partition in five elements. A transient, border-type partition appears also between region 3 and 4. The results are quite encouraging. We can progress thus further and test the algorithm on real geographical data.

### Results for real geographical data

In order to apply the method on real-life data we need a delimited geographical space for which there is settlement level data available for a relevant socio-economic measure on a long time-period. The GPS coordinates of settlements are usually well-known. We have found freely available data on the Internet for U.S.A on county level (GPS coordinates [37] and population census data for the last 5 census, [38]), and for Transylvania on settlement level (GPS coordinates [39] and population census data for 11 census between 1850 to 2002 [40]). It is quite probable that data for other geographical territories are also freely available on the Internet. The group of Professor József Nemes-Nagy from the Eötvos Lorand University of Budapest (Hungary) provided us an excellent data for the settlements in Hungary. This data includes the GPS coordinates of all settlements and the average local tax payed per inhabitant for the last 20 years (1990–2009). Among all these datasets the data for Hungary is the most complete one. Moreover, this is our only dataset which considers a financial measure. We expect thus that this data can help us in revealing administrative partitions, since financial aspects are centralized in Hungary on administrative (county) level. The data for USA and Transylvania concerns population sizes of counties and settlements, respectively. Such data will reflect especially historic regions, and are not useful to detect an administrative partition. Moreover, the data for USA is quite poor, since it reflect only five consecutive census years. There is thus week hope, that the data for USA can lead to an interpretable region structure. Nevertheless, these datasets gave us the possibility to test the method on real system.

**Figure 6:**
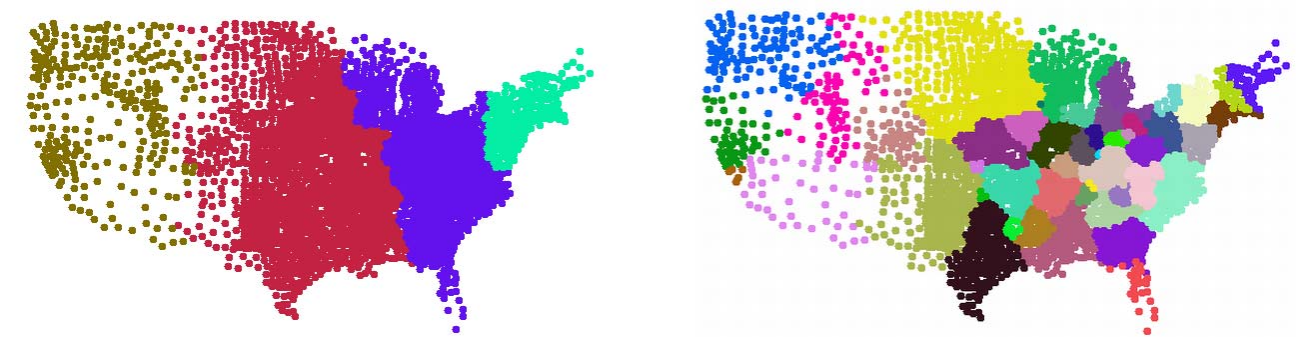
Results of the spring-block approach for USA using a five year census data. On the left we illustrate a rough partition in 4 and on the right we illustrate a finer partition in 48 elements.

In the case of U.S.A, partitions obtained on two different scales are presented on Figure 6. One can note the rough partition showing a division from east to west following the latitudes, and the finer partitions in 48 units (Alaska and Hawaii being not the subject of the analysis), revealing federal state-like structures. In agreement with the state map of USA we note the presence of smaller regions in the eastern part and large ones in the central and western part. Of course one cannot expect from this rough 5 year census data to regain the realistic state-map of the USA.

**Figure 7:**
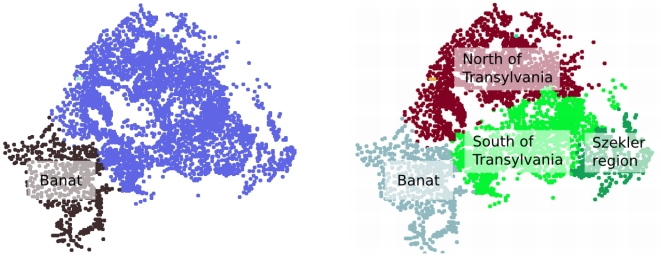
Results of the method for Transylvania using census data between 1850 and 2002. The picture on the left illustrates a rough partition, confirming that Banat is historically not belonging to Transylvania. The picture on the right shows a finer partition in four, revealing the main regions: north of Transylvania, south of Transylvania, Banat and the Szekler region.

In the case of Transylvania the rough partition in two reveals first the Banat region which is historically known to be different from the rest of Transylvania. The finer partition reveal other generally accepted regions like north of Transylvania, south of Transylvania, Banat and the Szekler region (Figure 7).

**Figure 8:**
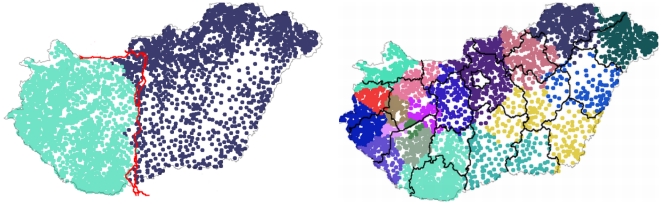
Results of the method for Hungary using settlement level tax data between 1990 and 2009. The picture on the left illustrates a first partition, revealing East and West Hungary separated by the line of the Danube river (plotted here with red). The picture in the right shows a partition in 19 elements, and on this picture we also illustrate with black lines the real county level administrative division of Hungary (data regarding the line of the Danube and the county borders acquired from [43]).

For the case of Hungary a partition in two reveals the line of the Danube river which separates the country in East and West Hungary (left on Figure 8). A major success of the present method is that the line of the Danube appears naturally, although such geographical data was not introduced in the model. A finer partition in 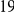 elements (the same as number of counties in Hungary) reveals groups of settlements which resembles the real county borders (Figure 8,right).

## Discussion

A simple method aimed for an objective partition of a geographical territory in region-like structures was presented. The method is motivated by a classical model of physics, namely the spring-block system. Our approach has several advantages: (1) it is an objective method, and it does not rely on adjustable parameters; (2) the involved analogies in defining the elements of the model are straightforward ones; (3) it works with different type of relevant data (population size, GDP, tax per inhabitant…), the only condition for them is to be available on settlement level and for a longer time-period; (4) it takes into account both spatiality and similarities of the neighboring settlements in forming the region-like structures; (5) it gives the partition on different scales; (6) it is easy to implement in a user-friendly graphical environment. To illustrate the applicability of the method, a user-friendly and interactive JAVA program was created and used for detecting region-like structures in the case of USA, Hungary and Transylvania. In the case of USA and Transylvania, long-term population census data was considered for constructing the connectivity measure, while in the case of Hungary taxation data for the last 20 years (1990–2009) were used. All these applications showed that the method works well, and the obtained space partitions proved to be reasonable ones, revealing some historically, politically or geographically motivated region-structures which correlates to a certain degree to the historical and geographical regions indicated in the standard literature [41], [42].

Although the results are promising ones, the potential of this method has to be weighted and analyzed with much care. One cannot state in any case that the proposed method solves the problem of defining and delimiting regions. Regional scientist and geographers will warn us [11], [12] that in order to do this one cannot work simply with a very limited number of objective measures, and usually there are many additional important and non-quantifiable parameters that have to be taken into account. Using one or two quantifiable parameters and incorporating them in a physical model is insufficient for giving a final answer in most of the cases. Such models and methods have to be used thus with consideration, and one has to realize their limitations. Nevertheless, objective space-partitioning methods are important and have their role in the process of defining regions. For example, if one uses the presented method with connectivity measures that reflects well all the relevant socio-economic interactions, the obtained space-partition agrees with the one suggested by social and regional studies. Our applications for the considered method proved this. Such methods can give thus for regional scientist, economist and politicians an extra confidence in their decisions and can help in solving endless disputes that are based on subjective interpretations. We consider thus the presented method useful in such sense.
